# Immunogenicity and functional evaluation of iPSC-derived organs for transplantation

**DOI:** 10.1038/celldisc.2015.15

**Published:** 2015-07-07

**Authors:** Libin Wang, Jiani Cao, Yukai Wang, Tianshu Lan, Lei Liu, Weixu Wang, Ning Jin, Jiaqi Gong, Chao Zhang, Fei Teng, Guoliang Yan, Chun Li, Jiali Li, Haifeng Wan, Baoyang Hu, Wei Li, Xiaoyang Zhao, Zhongquan Qi, Tongbiao Zhao, Qi Zhou

**Affiliations:** 1 State Key Laboratory of Reproductive Biology, Institute of Zoology, Chinese Academy of Sciences, Beijing, China; 2 Graduate University of Chinese Academy of Sciences, Beijing, China; 3 Organ Transplantation Institute, Xiamen University, Xiamen, China

**Keywords:** iPSC-derived organs, immunogenicity, transplantation

## Abstract

Whether physiologically induced pluripotent stem cell (iPSC)-derived organs are immunogenic and can be used for transplantation is unclear. Here, we generated iPSC-derived skin, islet, and heart representing three germ layers of the body through 4n complementation and evaluated their immunogenicity and therapeutic efficacy. Upon transplantation into recipient mice, iPSC-derived skin successfully survived and repaired local tissue wounds. In diabetic mouse models, explanted iPSC-derived islets effectively produced insulin and lowered blood glucose to basal levels. iPSC-derived heart grafts maintained normal beating for more than 3 months in syngeneic recipients. Importantly, no obvious immune rejection responses against iPSC-derived organs were detected long after transplantation. Our study not only demonstrates the fundamental immunogenicity and function of iPSC derivatives, but also provides preclinical evidence to support the feasibility of using iPSC-derived skin, islet, and heart for therapeutic use.

## Introduction

Induced pluripotent stem cells (iPSCs) are derived by ectopic expression of four transcription factors in somatic cells [1]. iPSCs exhibit transcriptional and epigenetic characteristics that are highly similar to those of embryonic stem cells (ESCs). They can undergo self-renewal and maintain pluripotency to differentiate into any cell type [2–6]. Thus, iPSCs hold great promise by providing unlimited autologous cells for regenerative medicine. The therapeutic potential of iPSCs has been extensively evaluated by transplantation of cells differentiated from iPSCs *in vitro* into different animal models of disease. For example, transplanted iPSC-differentiated cells can effectively treat sickle cell anemia, diabetes, Parkinson's disease, spinal cord injury, myocardial infarction, and so on [7–14]. However, a few studies have reported tumors were formed in iPSC-differentiated cells due to the low purity of differentiated cells and/or unstable *in vitro* differentiation systems [15–19]. Furthermore, some cells derived from mouse iPSC have been shown to elicit antigen-specific immune rejection responses in a teratoma formation model [20]. By transplantation of differentiated iPSC derivatives, recent studies demonstrated that myocardial and endothelial cells, but not hepatocytes, skin, bone marrow, or neuronal cells, induced immune rejection responses [21–23]. Yet, whether organs derived from iPSCs *in vivo* are immunogenic and can be safely used for transplantation is still unknown.

Here we established different integration-free iPSC lines and generated iPSC mice by 4n complementation. By transplanting organs isolated from 4n complementation mice into syngeneic recipient mice, we evaluated the survival, 'ground' immunogenicity, and potential therapeutic effects of these physiological iPSC derivatives. iPSC-derived skin, heart, and islet were used to represent the three germ layers of the body and transplanted into different mouse models. The ability of iPSC-derived skin explants to heal skin lesions was examined using a wound healing mouse model, while the effect of iPSC-derived islet on high blood glucose concentrations was assessed in diabetic mice. Vascularized heterotropic transplantation of iPSC-derived heart was also performed. Tumor formation, necrosis, and T-cell infiltration of each graft type was assessed at different time points after transplantation to specifically evaluate survival and immunogenicity of grafts.

## Results

### Generation of integration-free iPSC lines and tetraploid (4n) complementation mice

**Figure 1:**
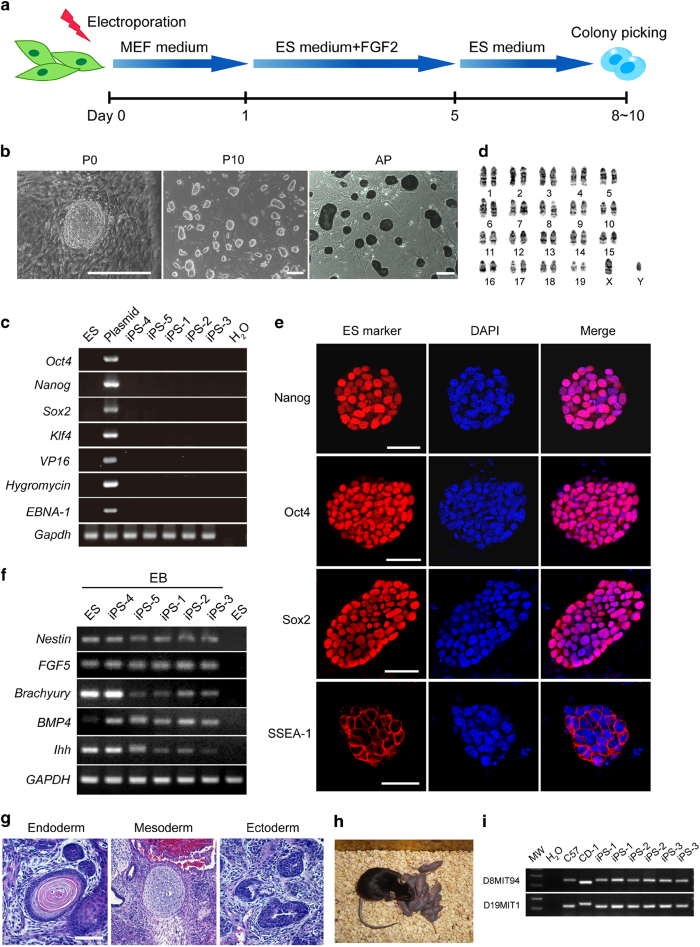
Generation of 4n complementation mice using integration-free induced pluripotent stem cells (iPSCs). (**a**) Integration-free iPSC generation method. (**b**) Morphology and alkaline phosphatase (AP) staining of integration-free iPSCs. Scale bars, 100 μm. (**c**) PCR analysis using seven primers spanning the whole episomal plasmid did not detect integration of the reprogramming vector in iPSm lines 1–5. The reprogramming plasmid was used as a positive control. (**d**) G-banding chromosomal analysis showed iPSCs had normal karotypes. (**e**) Immunofluorescence of pluripotent markers (Nanog, Oct3/4, Sox2, and SSEA-1) for iPSCs. (**F**) RT-PCR analysis showed iPSCs were well-differentiated into three germ layers. (**g**) iPSCs generated teratomas containing all three embryonic germ layers in severe-combined-immune-deficiency mice. (**h**) iPSC mice generated by 4n complementation. (**i**) SSLP analysis of mice derived from different iPSC lines.

Reactivation of integrated genomic reprogramming factors has been previously demonstrated and shown to contribute to tumorigenicity and immunogenicity of iPSC derivatives upon differentiation, which cannot represent the 'ground' characters of iPSC derivatives [2, 20, 24]. To rule out the effects of exogenous gene integration on intrinsic characteristics of iPSCs, we generated non-integrating iPSCs by delivering reprogramming factors into C57BL/6 mouse embryonic fibroblasts (MEFs) with an episomal vector (Figure 1a) [25]. The established iPSCs were alkaline phosphatase-positive and exhibited a cellular morphology similar to that of mouse ESCs (Figure 1b). PCR and southern blotting demonstrated that the iPSCs were free of plasmid integration (Figure 1c and Supplementary Figure S1A), had normal karotypes (Figure 1d), and expressed pluripotent genes (Figure 1e and Supplementary Figure S1B). Gene expression profiling indicated that global gene expression of iPSC lines (iPS-1, iPS-2, and iPS-3) was not obviously different from ESCs (Supplementary Figure S1D). Bisulfite sequencing showed that Oct4 and Nanog promoters of iPSCs were largely demethylated compared with MEFs (Supplementary Figure S1C). The iPSCs could differentiate into embryoid bodies and teratomas *in vitro*, validating their pluripotency (Figure 1f and g). Furthermore, using 4n complementation, we obtained viable and fertile mice from integration-free iPSC lines with high efficiency, which was comparable to that of normal ESCs (Figure 1h and i, and Supplementary Table 1), indicating the totipotency of these iPSCs.

**Figure 2:**
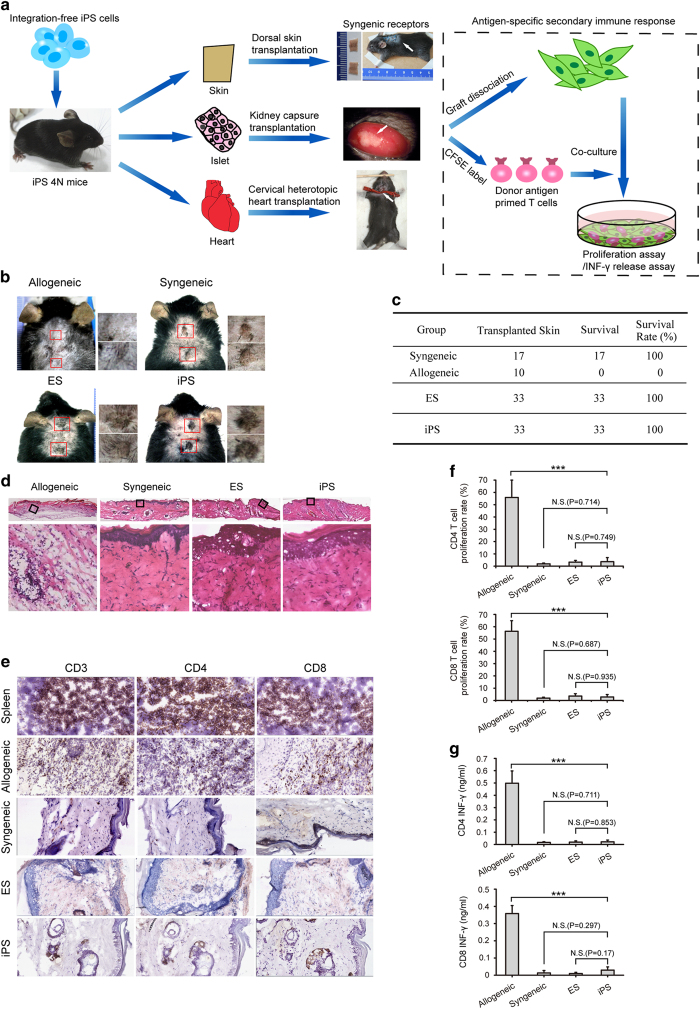
Induced pluripotent stem cell (iPSC)-derived skins were tolerated by hosts and successfully repaired skin wounds. (**a**) Schematic diagram of iPSm-derived organ transplantation. Skin, islets, and hearts dissociated from iPSC mice were transplanted onto the backs (top), under kidney capsules (middle), and on neck vessels (bottom), respectively. Primed T cells were detected by T-cell proliferation or interferon (INF)-γ release assays. (**b**) Wound repair by transplantation of iPSm-derived skin. Like ESm and syngeneic skin, iPSm skin effectively survived on recipient mice for more than 100 days after transplantation. Allogeneic skin transplants were rejected within 3 weeks and served as negative controls. Representative images show grafts 20 weeks after transplantation. (**c**) Survival rate summary of explanted iPSm skin 20 weeks after transplantation. ESm and syngeneic skin transplants served as positive controls; allogeneic skin transplants served as negative controls. (**d**) H&E staining of iPSm skin isolated from recipient mice 8 weeks after transplantation. Allografts were stained 1 week after transplantation and served as a negative control. As with ESm and syngeneic mice, iPSm skin explants showed normal structures, while extensive tissue necrosis was detected in allografts. (**e**) T-cell infiltration was hardly detected in iPSm skin explants 8 weeks after transplantation. T cells were identified by immunostaining with anti-CD3, anti-CD4, and anti-CD8 antibodies. Sections from the spleen and allogeneic skin grafts (1 week after transplantation) were used as positive controls. (**f**) The percentage of proliferating cells (low carboxyfluorescein succinimidyl ester) was quantified and shown as mean±s.e.m. of triplicates (syngeneic: *n*=3; ESm: *n*=6; iPSm: *n*=6; allogeneic: *n*=3). (**g**) IFN-γ release assay to detect primed T cells in iPSm skin recipients. The IFN-γ release was quantified and shown as mean±s.e.m. of triplicates (syngenic: *n*=3; ESm: *n*=6; iPSm: *n*=6; allogenic: *n*=3).

To evaluate the fundamental immunogenicity and function of iPSC derivatives for transplantation, inbred C57BL/6 mice derived from iPSC lines and ESC lines by 4n complementation were used as organ donors. Skin, islets, and heart were chosen as representatives of the three germ layers of the body (Figure 2a and Supplementary Table S1) to evaluate immunogenicity and function.

### Successful wound repair by iPSC-derived skin transplantation

To test the 'ground' function and immunogenicity of iPSC-derived skin, we performed a skin transplantation assay using a traditional 'tail-to-back' model (Figure 2a and Supplementary Figure S2A), which enables grafts to be distinguished from recipient tissue. Skin samples from the tails of iPSC-derived, ESC-derived, C57BL/6, and allogeneic mice were transplanted onto the backs of wounded C57BL/6 mice. Transplanted skin from iPSm (mice derived from iPSC lines by 4n complementation), ESm (mice derived from ESC lines by 4n complementation), and syngeneic mice successfully survived over 4 months and grew well and even regenerated hairs (Figure 2b and c). In contrast, all transplanted allogeneic skin samples elicited intensive rejection by recipient mice immediately after transplantation and were completely rejected within 2 weeks (Figure 2b–d, Supplementary Figure S2B and S2C).

To understand the successful transplantation of iPSm skin, skin grafts were isolated and stained with anti-CD3, anti-CD4, and anti-CD8 antibodies 3 months after transplantation. No obvious T-cell infiltration, tissue necrosis, or tumors were detected in iPSm, ESm, or syngeneic mouse skin explants. In contrast, explanted allogeneic skin showed extensive T-cell infiltration accompanied by serious tissue necrosis within 1 week after transplantation (Figure 2d and e). To further investigate whether iPSm skin could elicit antigen-specific immune rejection responses, secondary T-cell proliferation and IFN-γ release assays were performed. Splenic T cells were isolated from mice that received iPSm, ESm, syngeneic, and allogeneic skin, labeled with carboxyfluorescein succinimidyl ester, and re-exposed to cells isolated from explanted skin for 72 h. Compared with the prominent immune rejection response elicited by allogeneic grafts, substantially less T-cell proliferation was detected with iPSm, ESm, or syngeneic skins (Figure 2f, Supplementary Figure S2D and S2E). Consistent with T-cell proliferation, IFN-γ production was markedly stimulated by allogeneic but not syngeneic, iPSm, or ESm skins, indicating limited antigen-primed T cells in iPSm skin recipients (Figure 2g).

### Reduction of systematically high glucose by iPSC-derived islet transplantation

**Figure 3:**
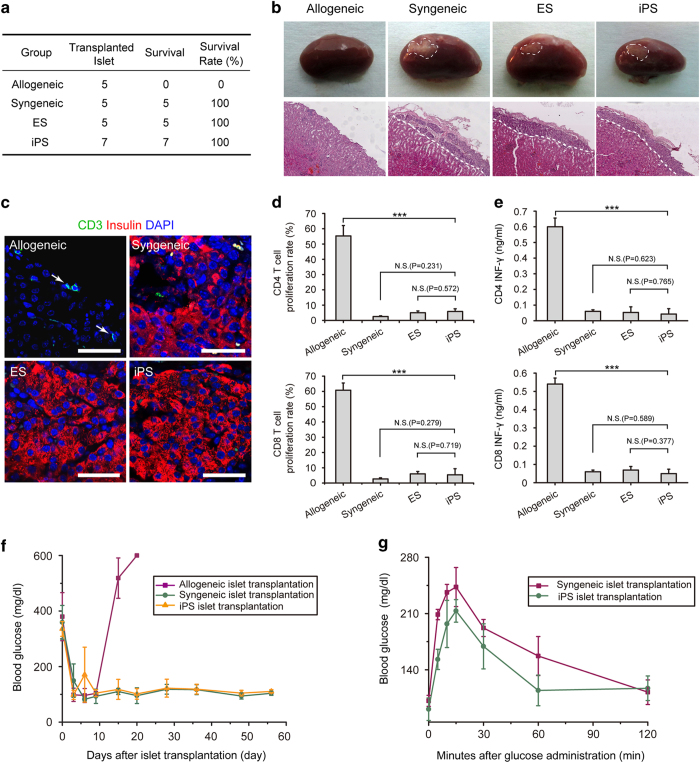
Induced pluripotent stem cell (iPSC)-derived islets reduced systematically high glucose in diabetic mice with limited immunogenicity. (**a**) Summary of iPSm islet survival in C57BL/6 hosts 8 weeks after transplantation. (**b**) Representative images of iPSm islets transplanted under kidney capsules (dot circles indicate grafted islets). (**c**) Detection of T-cell infiltration in iPSm islets with an anti-CD3 antibody (green). Anti-insulin staining was used to label engrafted islets (red). Scale bars, 50 μm. (**d**) T-cell proliferation induced by different stimulators was quantified and shown as the mean±s.e.m. (syngeneic: *n*=3; ESm: *n*=3; iPSm: *n*=3; allogeneic: *n*=3). (**e**) Interferon (IFN)-γ release was quantified and shown as mean±s.e.m. (syngeneic: *n*=3; ESm: *n*=3; iPSm: *n*=3; allogeneic: *n*=3). (**f**) Blood glucose levels were monitored in diabetic mice engrafted with allogeneic, syngeneic, and iPSm islets (iPSm: *n*=2, yellow; syngeneic: *n*=3, green; allogeneic: *n*=3, purple). (**g**) Glucose tolerance test 8 weeks after islet transplantation. Diabetic mice engrafted with iPSm islets (*n*=2, green) efficiently responded to high-glucose injection similar to mice transplanted with syngeneic islets (*n*=3, purple).

iPSm, ESm, C57BL/6, and Balb/c mouse islets were transplanted under the kidney capsule of C57BL/6 recipient mice to investigate their effects on blood glucose and immunogenicity (Figure 2a). Eight weeks after transplantation, all iPSm islet grafts survived in the recipients, whereas none of the allogeneic islets survived over 2 weeks (Figure 3a). Hematoxylin and eosin (H&E) staining of kidney grafts showed that iPSm, ESm, and syngeneic islet structures were well-maintained with no necrosis or tumor formation, while explanted allogeneic islets were completely rejected without any residues within 2 weeks (Figure 3b). Sections from explanted ESm, iPSm, allogeneic, and syngeneic islets were also stained with an anti-CD3 antibody. Compared with allogeneic islets that showed apparent T-cell infiltration, no obvious T-cell infiltration was detected in iPSm, ESm, and syngeneic islets 2 months after transplantation (Figure 3c and Supplementary Figure S3D). To further confirm the immunogenicity of iPSC-derived islets, secondary T-cell response and IFN-γ release assays were performed. Consistent with T-cell infiltration assays, allografts stimulated T-cell proliferation and IFN-γ production markedly, while iPSm, ESm, and syngeneic islets elicited limited responses (Figure 3d and e, and Supplementary Figure S3E).

To investigate the functional integrity of physiologically derived iPSm islets, we transplanted iPSm islet into diabetic mice. Intriguingly, 2 days after transplantation, the blood glucose of all diabetic recipients transplanted with iPSm and syngeneic islets decreased markedly to basal levels and maintained normal levels for more than 2 months (Figure 3f). Although the blood glucose concentration of diabetic mice treated with allogeneic islets significantly decreased 3–10 days after transplantation, levels increased 15 days after transplantation due to rejection of allogeneic islets (Figure 3c and f). Furthermore, iPSm islet recipients responded to glucose injections normally, and transplanted iPSm islets maintained normal structures 2 months after transplantation, indicating the limited immunogenicity of iPSC-derived islets did not affect grafting or functional integrity (Figure 3f and g; Supplementary Figure S3B and S3C). Taken together, these data support the clinical development and use of iPSC-derived islets for regenerative medicine.

### Grafted iPSC-derived hearts survived and maintained normal beating patterns in recipients

**Figure 4:**
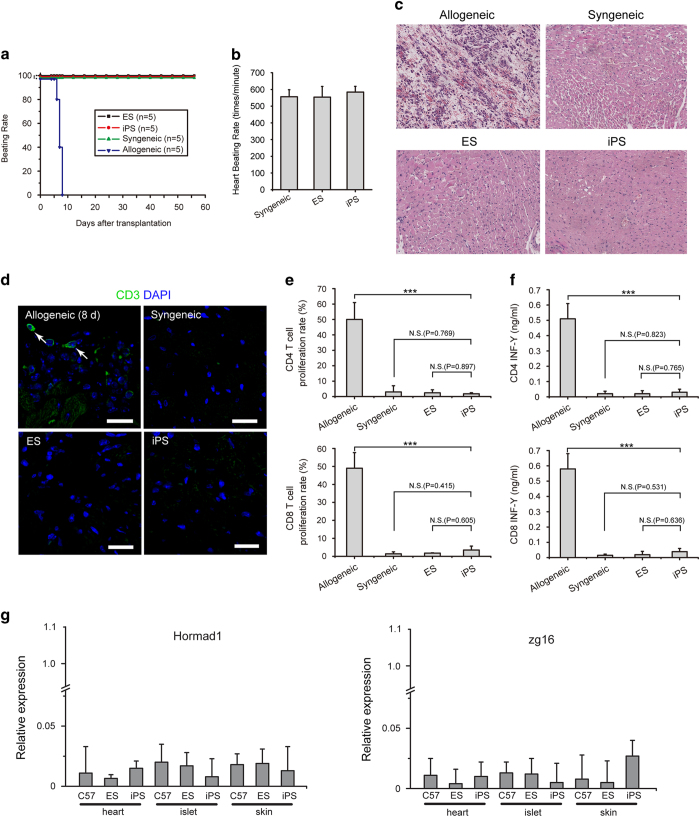
Induced pluripotent stem cell (iPSC)-derived heart transplantation. (**a**) Survival curve of iPSm, ESm, syngeneic, and allogeneic mouse hearts engrafted in C57BL/6 recipient mice (*n*=5). (**b**) Engrafted iPSm hearts showed similar beating rates as ESm and syngeneic mice. (**c**) H&E staining of transplanted hearts. (**d**) T-cell infiltration identified by staining sections with anti-CD3 antibodies (green). No obvious T-cell infiltration was detected in grafted iPSm, ESm, and syngeneic mouse hearts isolated from C57BL/6 recipients. Allografts showed extensive T-cell infiltration and served as positive controls. Scale bars, 50 μm. (**e**) T-cell proliferation and (**f**) interferon (INF)-γ release were used to detect the presence of primed T cells in C57BL/6 mice engrafted with iPSm, ESm, syngeneic, and allogeneic hearts (syngeneic: *n*=3; ESm: *n*=3; iPSm: *n*=3; allogeneic: *n*=3). (**g**) Expression of the *Zg16* and *Hormad1* genes in transplanted skin, islets and hearts 8 weeks after transplantation.

Vascularized heterotropic heart transplantation was performed to test the viability and immunogenicity of iPSC-derived hearts. Hearts isolated from iPSm, ESm, C57BL/6, and Balb/c mice were transplanted under the neck skin of C57BL/6 recipient mice via anastomosis to the neck vessels using a non-suture cuff technique described previously [26, 27] (Figure 2a). Similar to ESm and syngeneic heart transplants, iPSm hearts maintained normal beating patterns in recipients for over 2 months without any obvious defects, while all transplanted allogeneic hearts terminated beating within 10 days after transplantation (Figure 4a, b and Supplementary Figure S4A). Moreover, extensive necrosis and tissue regression were observed in allogeneic heart transplants. In contrast, transplanted iPSm, ESm, and syngeneic hearts maintained normal structures (Figure 4c), indicating the comparative fundamental function of iPSC-derived heart with that of syngeneic mice.

To test the intrinsic immunogenicity of iPSC-derived hearts and their potential effects on functional integrity, transplanted iPSm hearts were isolated and stained with an anti-CD3 antibody. Consistent with H&E staining, T cells were hardly detected in iPSm, ESm, and syngeneic heart explants, but had infiltrated allogeneic hearts (Figure 4d). T-cell proliferation and IFN-γ release assays further confirmed that transplanted iPSm hearts did not induce significant immune rejection responses (Figure 4e and f), indicating limited immunogenicity. Taken together, these data indicated that the limited immunogenicity of iPSm hearts did not affect their function, supporting the clinical application of iPSC-derived hearts in the treatment heart disease, failure, and so on.

## Discussion

Recent studies have shown certain cells (for example, cardiomyocytes and endothelial cells) differentiated from iPSCs *in vitro* can elicit immune rejection responses, possibly due to differential presentation of abnormalities induced by reprogramming upon iPSC differentiation [15, 20–23, 28]. However, whether physiologically derived iPSC progeny are immunogenic is relatively unknown. By transplantation of iPSC-derived skin, islet, and heart, we have shown that *in vivo* iPSC-derived organs can be accepted by the recipient immune system without the need for immunosuppression. In particular, we did not observe extensive infiltration or priming of T cells in mice transplanted with iPSC skin, islet, or heart, indicating limited immunogenicity of these organs upon transplantation. The expression of Hormad1 and Zg16 was not significantly increased in the iPSC skin, islet, or heart (Figure 4g). Previously, the Abe group reported that iPSC-derived skin and bone marrow cells obtained from chimeric mice generated by B6 iPSCs have negligible immunogenicity in the host and are completely immune-tolerated by B6 recipients [21]. However, iPSC-derived tissues from chimeric animals may be able to adapt their immunogenicity to the chimeric host immune system, thereby misrepresenting their intrinsic character. It is possible that abnormal or immunogenic iPSC-derived cells were immune-rejected and/or rendered immune-tolerant by the intrinsic immune system of chimeric B6 mice long before transplantation into other B6 recipients. In contrast to tissues derived from chimeric mice, those isolated from 4n complementation iPSC mice do not have these issues and represent the fundamental nature or 'ground state' of iPSC derivatives.

Organ transplantation has been widely used in clinics to replace damaged organs like the skin, kidney, heart, pancreas and so on. However, the severe shortage of donor organs and immune rejection are two major obstacles to the expansion of organ transplantation therapies. iPSCs hold great promise in regenerative medicine by potentially providing unlimited autologous resources without concerning on immune rejection. Significant progress has been made in using iPSCs for basic research and disease modeling; however, clinic use of iPSCs is still in its preliminary stage [16, 29–31]. The incomplete differentiation of iPSCs *in vitro* not only limits the generation of transplantable cells, but also poses a serious teratoma risk due to remaining undifferentiated pluripotent stem cells [16, 30, 31]. *In vitro* generation of organs from a patient’s own pluripotent stem cells is an ideal strategy proposed to address these issues and an ultimate goal for regenerative medicine. For a long time, regeneration of functional organs is hindered by the complexity of organogenesis. Recent advance in the use of interspecific blastocyst complementation systems for *in vivo* generation of organs derived from donor pluripotent stem cells is a very promising strategy to address this issue [32, 33]. In the meantime, regeneration of functional organs from adult tissue stem cells or iPSCs has enabled significant advancements in the generation of functional organs for regenerative medicine [34–38]. Given those significant progresses, however, whether iPSC-derived organs can be used for transplantation in theory is still largely unknown. In this context, the current study represents a ‘proof-of-principle’ preclinical experiment evaluating the potential feasibility of using iPSC-derived organs for transplantation.

Another major concern for clinical use of iPSCs is safety. Development of new reprogramming techniques (for example, integration-free and small-molecule induction) significantly reduces the potential cancer risks associated with viral integration [15, 39]. Meanwhile, studies have shown that reprogramming itself induces both genomic and epigenetic instability [2, 20, 24, 40–44]. Although studies using *in vitro* iPSC derivatives to treat disease models have shown promising clinical results [45], the therapeutic efficacy and safety of physiologically derived iPSC organs have never been explored. Encouragingly, in current study we did not detect tumorigenesis in or around the explanted iPSC-derived organs in our experimental settings. These data support the efficacy and safety of iPSC-derived skin, heart, and islet for therapy.

## Materials and Methods

### Generation of iPSCs with episomal plasmids

For reprogramming, 6 μg of episomal plasmids containing *Oct4*, *Klf4*, *Sox2*, *and Nanog*, which were generously provided by Dr. Guoliang Xu [25] were transfected into 1×10^6^ MEFs by a neon (Invitrogen, Carlsbad, CA, USA). Transfected MEFs were plated onto 2 mm×100 mm dishes pre-seeded with a layer of feeder cells. One day after transfection, the MEF medium was replaced with our previously reported ESC medium [6] plus 5 μg ml^−1^ FGF2 for the first 4 days; the culture medium was changed every other day. Colonies with morphology similar to ESCs appeared approximately 8 days after transfection and were picked ~10 days post transfection.

### PCR and southern blot analysis

Primers spanning the whole plasmid were used to detect reprogramming vector integration (Supplementary Table S2). For southern blot, 15 μg of genomic DNA was digested with *Eco*R V (Takara, Japan), electrophoresed on a 0.7% agarose gel overnight, and then transferred onto a positively-charged nylon membrane (Millipore, Darmstadt, Germany). Hybridization was performed at 67 °C with P^32^ radioactively labeled cAMP prepared by Prime-a-Gene Labeling System (Promega, Fitchburg, WI, USA). The cDNA of *Oct4*, *Nanog*, *Sox2*, and *Klf4* were used as probes (Supplementary Table S2).

### Karyotype analysis

Karyotyping was conducted using standard chromosomal analysis protocols. G-binding karyotype analysis was carried out by Peking Union Medical College (Beijing, China).

### Immunofluorescence and alkaline-phosphatase staining

Immunostaining was carried out as previously described [6]. In brief, samples were fixed with 4% paraformaldehyde for 30 min at ~25 °C then washed three times with PBS. Non-specific sites were blocked with 500 μl of 2% bovine serum albumin (BSA) plus 0.45% Triton X-100 for 1 h at 20–25 °C. The samples were then incubated with the primary antibody overnight at 4 °C. Primary antibodies included anti-Oct4 (Santa Cruz, Dallas, TX, USA, 1:50), anti-Sox2 (Millipore, 1:200), anti-SSEA1 (Millipore, 1:200), and anti-Nanog (Abcam, Cambridge, UK, 1:100). The next day, samples were washed three times with PBS, incubated with an AlexaFlur 488-conjugated secondary antibody (diluted in 2% BSA plus 4.5% Triton X-100) at 25 °C for 1 h, and then imaged with a LSM780 Meta confocal microscope (Zeiss, Oberkochen, Germany). An alkaline phosphatase staining kit (Beyotime, Shanghai, China) was used to detect iPSC colonies according to the manufacturer’s instructions.

### Bisulfite genomic sequencing

Bisulfite treatment of genomic DNA was performed with an EpiTect Bisulfite Kit (Qiagen) according to the manufacturer’s instructions. *Oct4* and *Nanog* promoter regions were amplified with nested primers as previously reported [6]. PCR was performed as follows: 94 °C for 5 min; 35 cycles at 94 °C for 30 s, 59 °C for 45 s, 72 °C for 30 min; and 72 °C for 7 min. PCR products were cloned into pMD18-T vectors (Takara), and 10 randomly selected clones were sequenced.

### Reverse transcriptase (RT)-PCR and qualitative (q)-PCR

Total RNA was extracted from tissues or cells using TRIZOL (Invitrogen). cDNA was synthesized with oligo (dT) primers (Promega) and M-MLV reverse transcriptase (Promega) according to the manufacturer’s instructions. RT-PCR was performed with ExTaq (Takara), and qPCR was performed with SYBR Green PCR master mixes (Toyobo, Osaka, Japan). Primers used are listed in Supplementary Table 2.

### Simple sequence length polymorphism analysis

The primers used for SSLP were selected from the Mouse Genome Informatics website (http://www.informatics.jax.org/). DNA was extracted from ears of mice. PCR products were separated by 3.5% agarose gels and visualized with ethidium bromide using a gel imager (Bio-Rad, Hercules, CA, USA).

### Teratoma formation

Approximately 1×10^7^ iPSCs were injected subcutaneously into the hind limbs of 10-week-old male severe-combined-immune-deficiency beige mice. Four weeks later, teratomas were disassociated, fixed with 4% paraformaldehyde, embedded in paraffin, sectioned, and stained with H&E for histological analysis.

### Tetraploid (4n) embryo complementation

Generation of mice by 4n embryo complementation was carried out as previously described [46] and handled according to CMPH and NIH guidelines. In brief, two-cell embryos were collected from oviducts of female CD-1 mice, subjected to electrofusion to produce one-cell 4n embryos, and then cultured in CZB media. Ten to 15 iPSCs were injected into each 4n blastocyst and transferred to CD-1 pseudopregnant recipient females. Embryos derived from 4n blastocyst injection were dissected at birth (embryonic day 19.5).

### Skin transplantation

Skin transplantation experiments were performed in accordance with the Chinese Ministry of Public Health Guide and US National Institutes of Health Guide for the Care and Use of Laboratory Animals. Male C57BL/6 mice (8 week-old, H2 haplotype: b) were anesthetized by intraperitoneal injection of sodium pentobarbital (40 mg kg^−1^ body weight). Then skin samples (8 mm×8 mm) from the tails of iPSC-derived (*n*=33), ESC-derived (*n*=33), C57BL/6 (*n*=17), or 129SVJ (H2 haplotype: bc) (*n*=10) mice were transplanted onto the back of C57BL/6 recipient mice.

### Diabetes mouse model and islet transplantation

Diabetes was induced by a single intraperitoneal injection of 180 mg kg^−1^ streptozocin (Sigma-Aldrich, St Louis, MO, USA) in 0.1 m citrate buffer (pH 4.4). Blood glucose was measured by a One-Touch Glucose Analyzer (FreeStyle, Abbott, Lake Forest, IL, USA). Mice with blood glucose concentrations above 16.7 mm for more than two consecutive days were used for transplantation. Donor islets were isolated from pancreases via bile duct cannulation with 3 ml collagenase P (1 mg ml^−1^; Roche, Penzberg, Germany) and digested for 20 min at 37 °C. The digested pancreatic tissues were washed and purified using an EZ-Sep lymphocyte separation kit (Dakewe Biotech, Guangzhou, China). Isolated islets were collected manually using a pipette under a microscope and injected under the kidney capsule of recipient mice (iPS group: *n*=7; ES group: *n*=5; syngenic group: *n*=5; allogenic group: *n*=5). Blood glucose in recipient mice was monitored every other day. Mice were fasted for 16 h before the glucose tolerance test, and blood glucose was measured before and 15, 30, 60, 90, and 120 min after glucose injection (2 g kg^−1^ weight glucose).

### Heart transplantation

Vascularized heterotopic heart transplantation was performed with anastomosis to the vessels of the neck using a non-suture cuff technique as previously described [26, 27] (*n*=7 each group). Cardiac graft survival was monitored by palpation daily for the first week, and every 3 days after. Recipient death and/or loss of graft function within 48 h after transplantation were considered as technical failures, and these animals were omitted from analysis.

### Histological analysis

Samples isolated from recipient mice were embedded in either OCT (ZLI-9302) or formalin, and then examined by H&E or immunohistochemical staining using anti-CD3 (1:200), anti-CD4 (1:500), anti-CD8 (1:200), and anti-insulin antibodies (BD Pharmingen, San Jose, CA, USA).

### Secondary T-cell response assay

CD4^+^ and CD8^+^ T cells were isolated from the spleens of recipient C57BL/6 mice grafted with iPSm skin, islets, and heart for 12 weeks using CD4^+^ and CD8^+^ T-Cell Isolation Kits (MACS 130-095-248; MACS 130-095-236). The isolated T cells (responders) were labeled with carboxyfluorescein succinimidyl ester (Sigma) and co-cultured with the cells digested from the transplanted grafts (stimulators) for 72 h. Proliferation of CD4^+^ and CD8^+^ T cells was analyzed by flow cytometry (Caliber, BD Pharmingen). carboxyfluorescein succinimidyl ester-stained CD4^+^ and CD8^+^ T cells without stimulation were used as a control. CD3-positive T cells were selected to calculate the proliferation of CD4^+^ and CD8^+^ T cells (*n*=3). Culture mediums were used to determine INF-γ release using an INF-γ ELISA kit (EMC101g.96; *n*=3) (Thermo, Waltham, MA USA).
