# Supplementary material for: Immunogenicity and functional evaluation of iPSC-derived organs for transplantation
Source: Cell Discov. 2015 Jul 7;1:15015–. doi: 10.1038/celldisc.2015.15 (PMC4860825; doi:10.1038/celldisc.2015.15)
Supplement: Supplementary Tables [file celldisc201515-s1.doc]

**Table S1. Developmental efficiency of embryos produced, related to Figure 1.**

| Cell type | Donor cells | No. of injected embryos | No. of implantations  (% injection) | No. of full-term pups  (% injection) | No. of survived  (% injection) |
| --- | --- | --- | --- | --- | --- |
| ES cells | ES-1 | 108 | 46 ( 42.6) | 17 (15.9) | 13 (12.0) |
|  | ES-2 | 153 | 61 (39.9) | 17 (11.1) | 9 (5.9) |
| Episome-derived iPS cells | iPS-1 | 118 | 51 (49.5) | 16 (13.6) | 10 (8.5) |
|  | iPS-2 | 173 | 90 (52.0) | 14 (8.1) | 8 (4.6) |
|  | iPS-3 | 108 | 49 (45.4) | 4 (3.7) | 3 (2.8) |

**Table S2. List of primers in our work, related to Figure 1, 2, 3, 4.**

| Gene | Primer | Sequence | Application |
| --- | --- | --- | --- |
| *Oct4-vp16* | F | AGCGACTATGCACAACGAGAGGA | Integration assay |
|  | R | GGCCATATCCAGAGCGCCGT |  |
| *Sox2-vp16* | F | GCTCTTGGCTCCATGGGTTCGG |  |
|  | R | TCGGCCATATCCAGAGCGCCG |  |
| *Nanog-vp16* | F | CTGCTGAGATGCCTCACACG |  |
|  | R | ACATTGGAAGGTTCCCAGTCG |  |
| *Klf4-vp16* | F | GTCTCTTCGTGCACCCACTT |  |
|  | R | ACGATCGTCTTCCCCTCTTT |  |
| *VP16* | F | CGGGATCCATGTTGGGGGACGGG |  |
|  | R | CGGGAATTCCCCACCG |  |
| *EBNA-1* | F | CACCCTCATCTCCATCACCT |  |
|  | R | AGTCGTCTCCCCTTTGGAAT |  |
| *Hygromycin* | F | GTGTCACGTTGCAAGACCTG |  |
|  | R | ACATTGTTGGAGCCGAAATC |  |
| *Oct4* | F | ATGGCGGGACACCTGGCTTC | Southern blot |
|  | R | TCAGTTTGAATGCATGGGAGAG |  |
| *Sox2* | F | ATGTACAACATGATGGAGAC |  |
|  | R | CATGTGTGAGAGGGGCAGTG |  |
| *Nanog* | F | ATGAGTGTGGATCCAGCTTG |  |
|  | R | CACGTCTTCAGGTTGCATG |  |
| *Klf4* | F | ATGAGGCAGCCACCTGGCGAGTC |  |
|  | R | AAAATGCCTCTTCATGTGTAAG |  |
| *Hormad1* | F | CCAGATTACCAACCACCAG | qPCR |
|  | R | TGAAAAGGTGTTGGGACT |  |
| *Zg16* | F | CATCACCGCCTTCCGTAT |  |
|  | R | CGTTGAAACTTGTGCCTGA |  |
| *oct4* | F | AGAGGATCACCTTGGGGTACA |  |
|  | R | CGAAGCGACAGATGGTGGTC |  |
| *Sox2* | F | GCGGAGTGGAAACTTTTGTCC |  |
|  | R | GGGAAGCGTGTACTTATCCTTCT |  |
| *Nanog* | F | CACAGTTTGCCTAGTTCTGAGG |  |
|  | R | GCAAGAATAGTTCTCGGGATGAA |  |
| *Klf4* | F1 | GGCGAGTCTGACATGGCTG |  |
|  | R1 | GCTGGACGCAGTGTCTTCTC |  |
| *Rex1* | F2 | CCGGGATGAAAGTGAGATTAGC |  |
|  | R2 | TCACCTCGTATGATGCACTCT |  |
| *Dppa3* | F1 | GACCCAATGAAGGACCCTGAA |  |
|  | R1 | GCTTGACACCGGGGTTTAG |  |
| *GAPDH* | F2 | AGGTCGGTGTGAACGGATTTG |  |
|  | R2 | GGGGTCGTTGATGGCAACA |  |
| *Nestin* | F | AGAGTCAGATCGCTCAGATCC | RT-PCR |
|  | R | GCAGAGTCCTGTATGTAGCCAC |  |
| *Brachyury* | F | GCTTCAAGGAGCTAACTAACGAG |  |
|  | R | CGTCACGAAGTCCAGCAAGA |  |
| *BMP4* | F | ATTCCTGGTAACCGAATGCTG |  |
|  | R | CCGGTCTCAGGTATCAAACTAGC |  |
| *Ihh* | F | GGCGCTACGAAGGCAAGAT |  |
|  | R | CTTGAAGATGATGTCGGGATTGT |  |
| *FGF5* | F | GAAGCGTCTCACTCCCGAAG |  |
|  | R | GAAGAAAACGTCGCGCTACT |  |
