# Supplementary material for: Immunogenicity and functional evaluation of iPSC-derived organs for transplantation
Source: Cell Discov. 2015 Jul 7;1:15015–. doi: 10.1038/celldisc.2015.15 (PMC4860825; doi:10.1038/celldisc.2015.15)
Supplement: Supplementary Figures [file celldisc201515-s2.pdf]

Figure S1

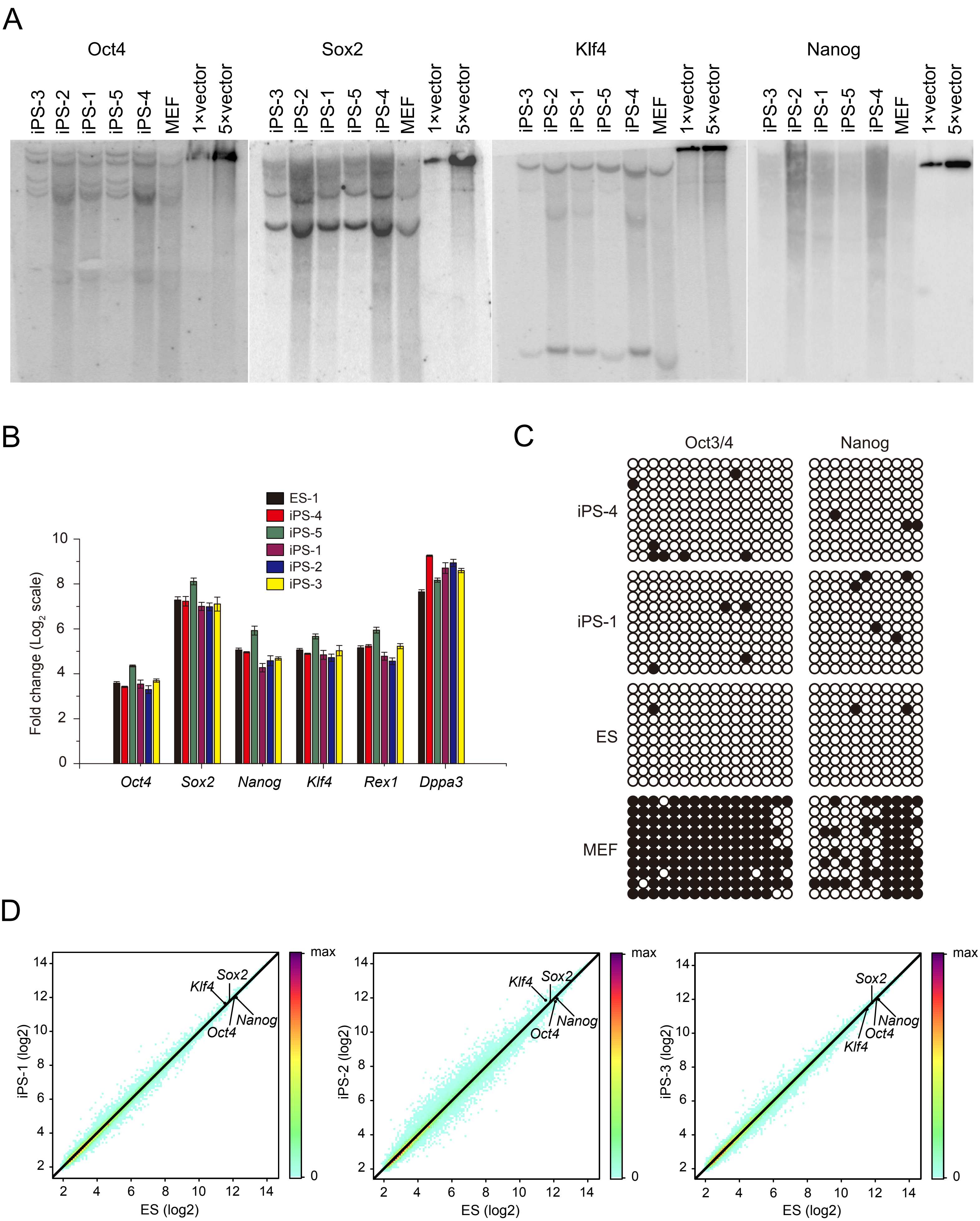

A
Oct4
Sox2
Klf4
Nanog
iPS-3
iPS-2
iPS-1
iPS-5
iPS-4
MEF
1×vector
5×vector
B
ES-1
iPS-4
iPS-5
iPS-1
iPS-2
iPS-3
Fold change (Log2 scale)
0
2
4
6
8
10
Oct4
Sox2
Nanog
Klf4
Rex1
Dppa3
C
Oct3/4
Nanog
iPS-4
iPS-1
ES
MEF
D
iPS-1 (log2)
iPS-2 (log2)
iPS-3 (log2)
ES (log2)
2
4
6
8
10
12
14
Klf4
Sox2
Nanog
Oct4
max
0

Figure S2

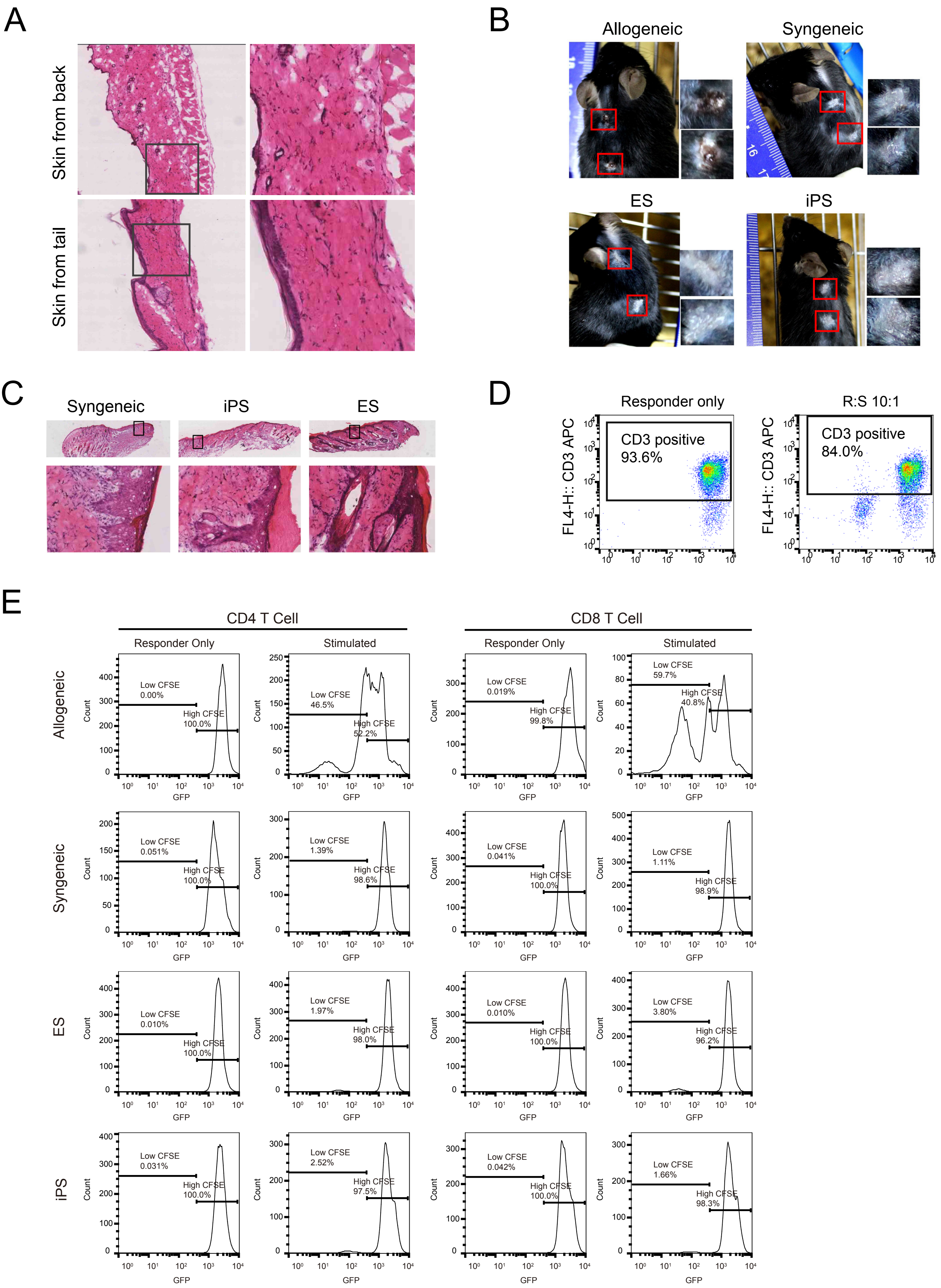
A
Skin from back
Skin from tail
B
Allogeneic
Syngeneic
ES
iPS
C
Syngeneic
iPS
ES
D
Responder only
R:S 10:1
FL4-H:: CD3 APC
CD3 positive
93.6%
CD3 positive
84.0%
E
CD4 T Cell
CD8 T Cell
Responder Only
Stimulated
Responder Only
Stimulated
Allogeneic
Syngeneic
ES
iPS
Count
GFP
Low CFSE
High CFSE
0.00%
100.0%
46.5%
52.2%
0.019%
99.8%
59.7%
40.8%
0.051%
100.0%
1.39%
98.6%
0.041%
100.0%
1.11%
98.9%
0.010%
100.0%
1.97%
98.0%
0.010%
100.0%
3.80%
96.2%
0.031%
100.0%
2.52%
97.5%
0.042%
100.0%
1.66%
98.3%

Figure S3

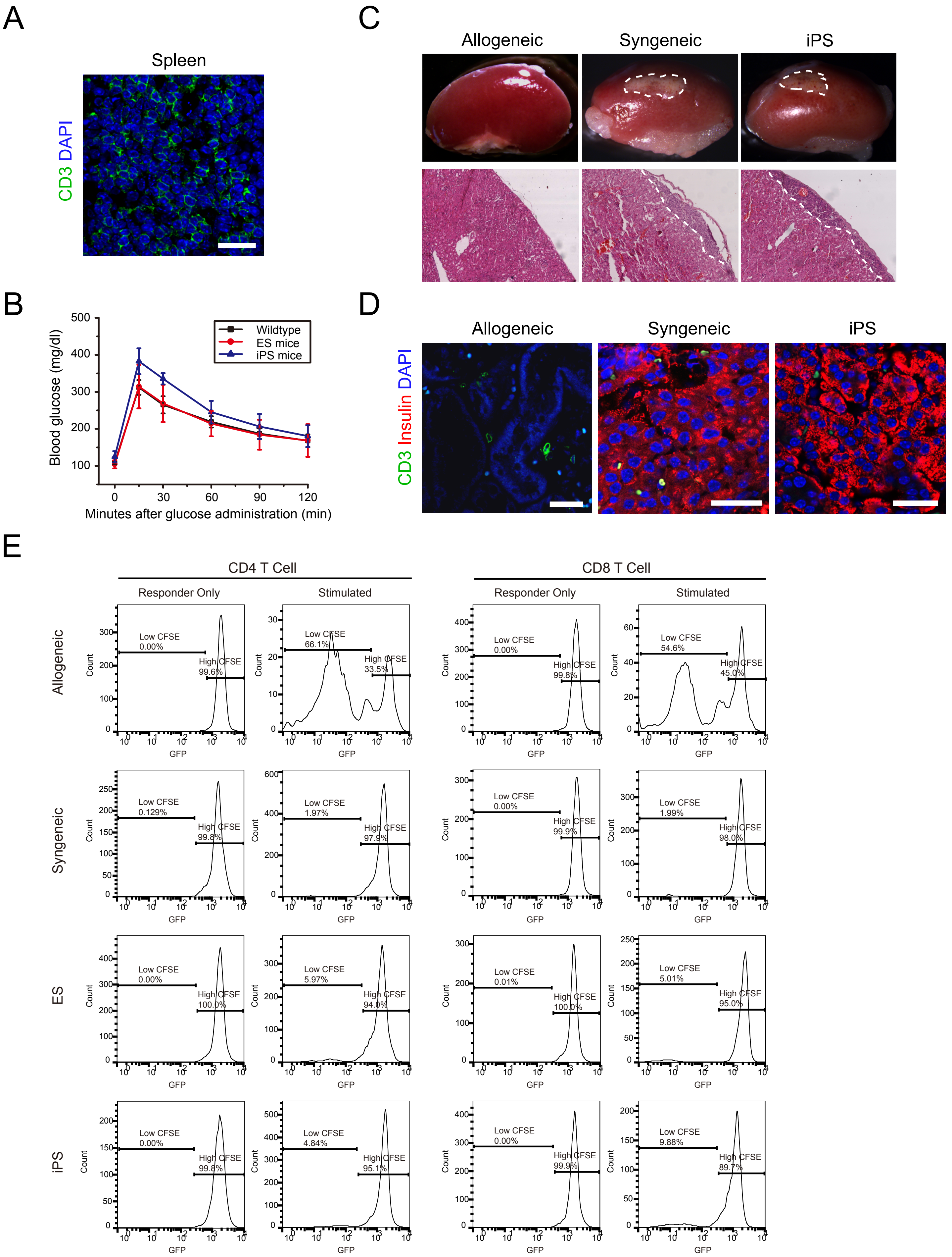

A
Spleen
CD3 DAPI
B
Wildtype
ES mice
iPS mice
Blood glucose (mg/dl)
Minutes after glucose administration (min)
C
Allogeneic
Syngeneic
iPS
D
Allogeneic
Syngeneic
iPS
CD3 Insulin DAPI
E
CD4 T Cell
CD8 T Cell
Responder Only
Stimulated
Allogeneic
Syngeneic
ES
iPS
Low CFSE
High CFSE
Count
GFP

Figure S4

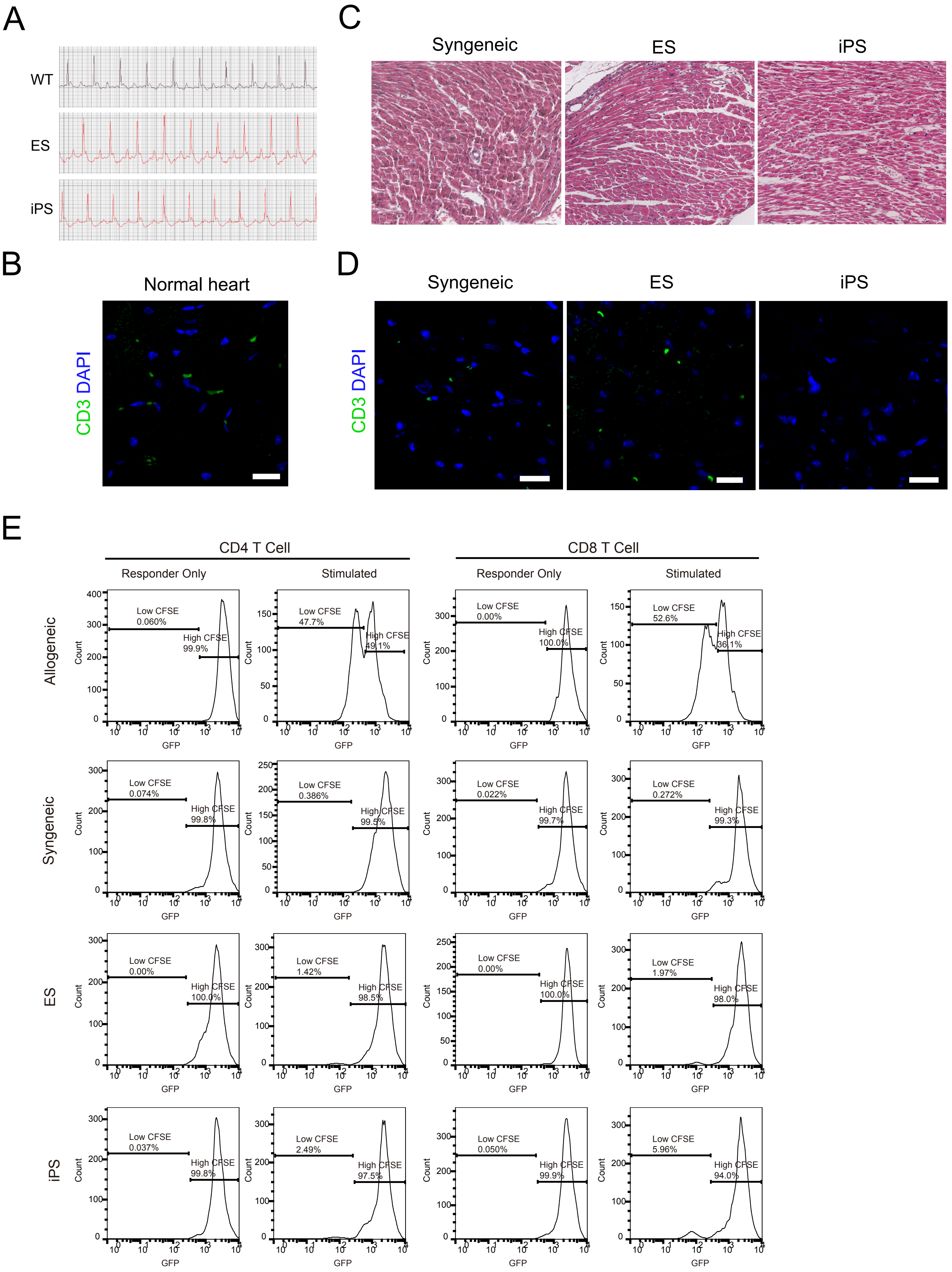

A
WT
ES
iPS
C
Syngeneic
ES
iPS
B
Normal heart
CD3 DAPI
D
Syngeneic
ES
iPS
CD3 DAPI
E
CD4 T Cell
CD8 T Cell
Responder Only
Stimulated
Responder Only
Stimulated
Allogeneic
Syngeneic
ES
iPS
Count
GFP
Low CFSE
High CFSE
0.060%
99.9%
47.7%
49.1%
0.00%
100.0%
52.6%
36.1%
0.074%
99.8%
0.386%
99.5%
0.022%
99.7%
0.272%
99.3%
0.00%
100.0%
1.42%
98.5%
0.00%
100.0%
1.97%
98.0%
0.037%
99.8%
2.49%
97.5%
0.050%
99.9%
5.96%
94.0%

Supplementary Figure Legends

Figure S1. Characterization of integration-free iPSCs.

(A) Southern blotting indicated no random integration of the episomal vector in iPSm-1, -2, -3, -4, or -5 lines. Genomic DNA extracted from these five iPSC lines and MEFs was digested with EcoR V and hybridized with Oct4, Sox2, Klf4, and Nanog cDNA probes. (B) qPCR analysis of endogenous pluripotent gene expression in the integration-free iPSC lines. (C) Methylation analysis of Oct4 and Nanog promoters by bisulfite genomic sequencing. Open and closed circles indicate unmethylated and methylated CpGs, respectively. ESCs and MEFs were used as positive and negative controls, respectively. (D) Scatter-plots compared expression values for all probe sets from genome-wide transcription profiling derived from ESm-1 and iPSm-3, -4, and -5 lines.

Figure S2. iPSC-derived skin transplantation.

(A) Different structure of tail and back skin. (B) Transplanted skin from iPSm, ESm, and syngeneic mice were accepted by recipients, while allogeneic skin elicited strong rejection 2 weeks after transplantation. (C) H&E staining of iPSm, ESm, and syngeneic skin engrafted to C57BL/6 recipients 2 weeks after transplantation. (D) For T-cell proliferation assay, $CD3^{+}$ cells were gated to analyze CFSE signals. (E) Fluorescence-activated cell signaling was used to detect T-cell proliferation stimulated by iPSm, ESm, syngeneic, and allogeneic skin cells.

Figure S3. iPSC-derived islet transplantation.

(A) Spleen sections were stained with anti-CD3 antibody (green) and served as a positive control. Scale bars, 50μm. (B) The glucose tolerance test was performed before iPSm islet transplantation; all tested mice showed a normal ability to lower blood glucose. Blood glucose levels were quantified and shown as mean ± SEM (n=3). (C) Representative images of transplanted iPSm islets under the subcapsular renal space of diabetic mice. Engrafted allogeneic islets were totally rejected within 4 weeks of transplantation, while explanted iPSm and syngeneic islets survived well in recipients 100 d after transplantation. (D) Anti-CD3 (green) and anti-insulin (red) staining of engrafted iPSm islets 100 d after transplantation. Scale bars, 50 μm. (E) Fluorescence-activated cell sorting was used to detect proliferation of $CD4^{+}$ and $CD8^{+}$ T-cells stimulated by iPSm, ESm, syngeneic, and allogeneic mouse islets.

Figure S4. iPSC-derived heart transplantation.

(A) Electrocardiography was used to test heart function before transplantation. (B) Normal hearts stained with anti-CD3 antibody. Scale bars, 50 μm. (C) Like ESm and syngeneic mice,

transplanted iPSm hearts showed normal structure 100 d after transplantation. (D) Limited T-cell infiltration was observed in iPSm and syngeneic hearts grafts 100 d after transplantation. T-cells were identified by anti-CD3 antibody. Scale bars, 50 μm. (E) Fluorescence-activated cell sorting was used to detect primed T-cells in mice transplanted with iPSm, ESm, syngeneic, and allogeneic hearts.
